# Phenotypic factors associated with amisulpride‐induced weight gain in first‐episode psychosis patients (from the OPTiMiSE cohort)

**DOI:** 10.1111/acps.13074

**Published:** 2019-07-19

**Authors:** R. Pandit, D. Cianci, S. E. ter Hark, I. Winter‐van Rossum, B. H. Ebdrup, B. V. Broberg, M. P. Garcia‐Portilla, J. Bobes, C. H. Vinkers, R. S. Kahn, S. Guloksuz, A. D. R. Huitema, J. J. Luykx

**Affiliations:** ^1^ Department of Translational Neuroscience Brain Center Rudolf Magnus University Medical Center Utrecht Utrecht University Utrecht the Netherlands; ^2^ Department of Biostatistics and Research Support Julius Center for Health Sciences and Primary Care University Medical Center Utrecht University of Utrecht Utrecht the Netherlands; ^3^ Department of Psychiatry Brain Center Rudolf Magnus University Medical Center Utrecht Utrecht University Utrecht the Netherlands; ^4^ Centre for Neuropsychiatric Schizophrenia Research CNSR & Centre for Clinical Intervention and Neuropsychiatric Schizophrenia Research, CINS Mental Health Centre Glostrup Copenhagen University Hospital Glostrup Denmark; ^5^ Department of Clinical Medicine Faculty of Health and Medical Sciences University of Copenhagen Copenhagen Denmark; ^6^ Department of Psychiatry and CIBERSAM University of Oviedo Oviedo Spain; ^7^ Instituto de Investigación Biosanitaria del Principado de Asturias (ISPA) Oviedo Spain; ^8^ Department of Psychiatry Amsterdam UMC (location VUmc) Amsterdam the Netherlands; ^9^ Department of Anatomy and Neurosciences Amsterdam UMC (location VUmc) Amsterdam the Netherlands; ^10^ Department of Psychiatry Icahn School of Medicine Mount Sinai New York USA; ^11^ Department of Psychiatry and Neuropsychology School for Mental Health Neuroscience Maastricht University Medical Center Maastricht the Netherlands; ^12^ Department of Psychiatry Yale School of Medicine New Haven CT USA; ^13^ Department of Pharmacy Pharmacology The Netherlands Cancer Institute Amsterdam the Netherlands; ^14^ Department of Clinical Pharmacy University Medical Center Utrecht Utrecht University Utrecht the Netherlands; ^15^ GGNet Mental Health Apeldoorn the Netherlands

**Keywords:** antipsychotic, weight gain, schizophrenia, amisulpride, psychosis

## Abstract

**Objective:**

Antipsychotic‐induced weight gain (AiWG) is a debilitating adverse effect of most antipsychotics. First‐episode psychosis patients are particularly vulnerable to the detrimental consequences of AiWG. Amisulpride has good efficacy and tolerability. We here aimed to identify the phenotypic factors associated with amisulpride‐induced weight gain in first‐episode psychosis patients.

**Method:**

Data were collected from the Optimization of Treatment and Management of Schizophrenia in Europe trial. Multivariable regression models with various phenotypic variables (*N* = 305) were performed with absolute AiWG and clinically relevant AiWG (≥7% AiWG) as outcomes.

**Results:**

Four weeks of amisulpride treatment increased body weight from 69.7 to 72.4 kg (*P* < 0.001). In the regression model of absolute AiWG, unemployment (*β* = 0.94, *P* = 0.016), younger age (*β* = −0.07, *P* = 0.031) and absence of current comorbid major depression disorder (*β* = −1.61, *P* = 0.034) were positively associated with absolute AiWG. In the regression model of clinically relevant AiWG, unemployment (OR = 2.83, *P* = 0.001), schizophreniform disorder (OR = 2.00, *P* = 0.025) and low baseline weight (OR = 0.97, *P* = 0.032) increased the likelihood of clinically relevant AiWG.

**Conclusions:**

Clinicians prescribing amisulpride should consider the relatively high susceptibility to AiWG in unemployed first‐episode patients with psychosis, in particular young subjects with a diagnosis of schizophreniform disorder. We advise to carefully monitor these patients and, when needed, implement weight‐reducing strategies.

**Summations**

- Variables associated with amisulpride‐induced weight gain are as follows: unemployment, absence of current comorbid depression, a diagnosis of schizophreniform disorder, young age and low baseline weight.
- Several phenotypic variables previously associated with antipsychotic‐induced weight gain were not found to be associated with amisulpride‐induced weight gain.

**Limitations**

- Although our sample size was fairly large and the association between amisulpride‐induced weight gain with unemployment was validated across models, a replication cohort was unavailable as the clinical trial where we drew our data from is unique in its kind.

## Introduction

Antipsychotic‐induced weight gain (AiWG) is one of the most common side‐effects of antipsychotics and is a well‐known risk factor for type 2 diabetes mellitus and metabolic syndrome 1. The prevalence of metabolic syndrome in patients treated with antipsychotics is almost twice that of unmedicated schizophrenia patients 2. AiWG also carries psychosocial consequences such as lower self‐esteem and social isolation which may subsequently trigger treatment non‐adherence 3. These consequences of AiWG not only negatively influence the effectiveness of antipsychotic therapy but they also affect quality of life and mortality in patients using antipsychotics 4. In fact, cardiovascular diseases as a result of metabolic syndrome are one of the leading causes of mortality in schizophrenia patients 4. Strikingly, first‐episode psychosis patients seem particularly vulnerable to AiWG 5. To date, several demographic and clinical factors have been associated with AiWG, most importantly young age, female sex, non‐white ethnicity, higher antipsychotic dose and lower BMI 6, 7, 8.

Amisulpride is a second‐generation dopamine receptor antagonist and has been shown to be one of the most effective first‐line treatment options in first‐episode schizophrenia patients 9. Both short‐ and long‐term treatments with amisulpride have been associated with clinically relevant AiWG (≥7% body weight change from baseline) in about one‐fifth of the patients 9, 10, 11. Although the weight gain figures following amisulpride use are lower compared to other second‐generation antipsychotics 12, for example weight gain due to clozapine or olanzapine can be as high as 50%, labelling amisulpride as a weight‐neutral drug seems unjustified 12. With few exceptions 13, the majority of studies investigating amisulpride‐induced weight gain have focused on long‐term treatment with amisulpride 12, 14, 15. Moreover, the influence of sociodemographic factors (age, sex, ethnicity) and dosing on amisulpride‐induced weight gain is poorly understood 8, possibly due to its labelling as a weight‐neutral drug. Furthermore, the effects of unemployment, a risk factor for weight gain in the general population 16, have to our knowledge not been studied in relation to AiWG. Economic uncertainty 16, 17 or passive lifestyle 18, 19 that are both associated with unemployment – can trigger weight gain through unhealthy food choices and decreased physical activity. Similarly, effects of additional factors such as baseline psychopathology and presence of comorbid major depressive disorder (MDD) that may also negatively affect food choices and thus stimulate weight gain have to the best of our knowledge not been investigated in this context either 17. Awareness about the sociodemographic and clinical factors associated with amisulpride‐induced weight gain will help clinicians identify patients at risk of AiWG, thus allowing them to monitor more closely and possibly treat AiWG.

### Aims of the study

To comprehensively dissect the phenotypic factors associated with amisulpride‐induced weight gain, we leveraged the size and homogeneity of the OPTiMiSE (Optimization of Treatment and Management of Schizophrenia in Europe) trial 9. We identified several variables that may be used in clinical practice to help clinicians monitor amisulpride‐induced weight gain more closely.

## Methods

### Clinical trial registration

The data analysed in the current study were obtained from the OPTiMiSE trial that is registered with ClinicalTrials.gov, number NCT01248195 9, 20. The authors assert that all procedures contributing to this work comply with the ethical standards of the relevant national and institutional committees on human experimentation and with the Helsinki Declaration of 1975, as revised in 2008. All procedures involving human subjects/patients were approved by the Medical Ethical Committee at the Sponsor site, University Medical Center Utrecht, the Netherlands, under registration number 11‐006/G‐E and NL34602.041.11. Regulatory approval was obtained in each individual country.

### Design, intervention and data collection

Details on study design and procedures, inclusion and exclusion criteria have been described previously 9. Briefly, first‐episode patients diagnosed with schizophrenia between 18 and 40 years of age were included in the trial. Patients with prior use of antipsychotics more than 2 weeks in the year before enrolment or 6 weeks lifetime were excluded. Written informed consent was obtained from all patients 20. Data were drawn from the first of three treatment phases of the OPTiMiSE trial: this was a 4‐week open‐label, single‐arm trial of treatment with amisulpride at the optimal dose (200–800 mg/day) was analysed in the current article. Nine patients receiving a dose higher than 800 mg/day were also included in the analyses. Study sites had been instructed to measure weight as consistently as possible across visits and subjects (e.g. using the same scale and having patients either wear or not wear clothes during measurements).

### Statistical analysis

Weight gain was defined as the difference in weight between the screening visit (a maximum of 1 week before initiation of phase I) and the end of the aforementioned phase I trial of amisulpride. Quantitative data were summarized as means and standard deviations (SD), while categorical variables were reported as counts with percentages. Change in body weight was tested using student's *t*‐test (alpha = 0.05). Our initial study population consisted of 320 patients (Table S1).

We ran a multivariable linear regression model with absolute body weight gain as outcome and a multivariable logistic regression model with clinically significant body weight gain as outcome and the following variables that were assessed at baseline.

#### Variables previously associated with AiWG

The variables, age, sex, race and dose, and prior antipsychotic exposure have been previously associated with AiWG 7, 8 and were included as predictor variables in both statistical models. Based on the available literature 7, we further included absolute antipsychotic naïveness (defined as no prior exposure to antipsychotics lifetime as reported by the participant) as a dichotomous variable into our statistical model given possible effects of any prior exposure (i.e. short duration of exposure: <2 weeks in the year before enrolment or <6 weeks lifetime). Likewise, effects of symptom severity as determined by PANSS (Positive and Negative Syndrome Scale) scores and treatment setting (in‐patient vs. out‐patient) were added to both statistical models. Diagnoses of schizophrenia spectrum disorder and current comorbid MDD were established according to the Mini International Neuropsychiatric Interview 5 plus 21.

#### Variables previously associated with weight gain in the general population

The variables employment status and comorbid MDD have been associated with weight gain in the general population 16, 17 and were therefore included in the current analyses.

#### Additional variables

We included diagnostic subtype of schizophrenia spectrum disorder as a proxy for disease severity/duration and treatment setting (in‐patient vs. out‐patient) as a proxy for compliance in both statistical models. For the diagnostic subtype variable, schizophrenia (*N* = 166) and schizoaffective disorder (*N* = 18) patients were pooled into one group given their similarities in duration of illness relative to schizophreniform disorder patients (*N* = 136).

Thus, multivariable linear regression analysis was performed to investigate the association between sociodemographic factors (age, sex, race and employment status), baseline diagnosis (schizophreniform vs. schizophrenia), PANSS total scores at baseline, presence of current comorbid MDD, and treatment‐related factors (treatment setting, average dose and prior antipsychotic exposure) with absolute body weight gain. Logistic regression was performed with the same variables to study the association between these factors and clinically relevant body weight gain (defined as body weight change of 7% from baseline values 12). Both statistical models were corrected for body weight at baseline. Due to missing data for the variable of current comorbid MDD, 15 subjects were excluded, resulting in 305 subjects available for the regression analyses (for the other variables, all data were complete). Results of the linear regression are reported as β coefficients with 95% confidence intervals (CI), and results of the logistic regression are reported as odds ratios (OR) with 95% CI. The level of statistical significance was set to *P* < 0.05, two‐sided. Statistical analyses were performed in SPSS version 23 (IBM Corp, Armonk, NY).

## Results

A total of 446 patients were enrolled in the trial, and 371 patients completed phase I of the study. We included data on body weight at inception and end of phase I, which comprised 320 patients (Table 1). Four weeks of amisulpride therapy led to a statistically significant increase in absolute body weight from 69.7 (14.3) at baseline to 72.4 (14.3) at end of phase I (*t*‐test, *P* < 0.001), with minimal differences in the degree of AiWG across sites (Fig. S1). Eleven per cent of normal weight (BMI 20‐25) patients at trial inception became overweight or obese at the end of phase I (Table S2).

**Table 1:** Characteristics of patients at baseline categorized on the basis of clinically relevant body weight gain. Clinically relevant body weight gain is defined as body weight change of 7% from baseline values. Data represented as mean (SD) or count

Factors	Participants with <7% body weight gain (*N* = 250)	Participants with ≥7% body weight gain (*N* = 70)	All participants (*N* = 320)
Sociodemographic variables			
Age (years)	26.5 (6.2)	25.1 (5.3)	26.2 (6.08)
Sex			
Women	71 (28.4%)	21 (30%)	92 (28.7%)
Men	179 (71.6%)	49 (70%)	228 (71.3%)
Race			
White	222 (88.8%)	63 (90%)	285 (89.1 %)
Other	28 (11.2%)	7 (10%)	35 (10.9%)
Employment status			
Unemployed	130 (52%)	50 (71.4%)	180 (56.3%)
Employed or student	120 (48%)	20 (28.6%)	140 (43.7%)
Clinical variables			
Primary diagnosis			
Schizophreniform disorder	96 (38.4%)	40 (58.1%)	136 (42.5%)
Schizophrenia	154 (61.6%)	30 (42.9%)	184 (57.5%)
Comorbid MDD†			
Yes	18 (7.6%)	2 (2.9%)	20 (6.6%)
No	218 (92.4%)	67 (97.1%)	285 (93.4%)
PANSS total scores‡	78.9 (19.0)	72.9 (17.7)	77.7 (18.9)
Treatment‐related variables			
Antipsychotic naïveness§			
Yes	107 (42.8%)	23 (32.9%)	130 (40.6%)
No	143 (57.2%)	47 (67.1%)	190 (59.4%)
Type of care at baseline			
In‐patient	155 (62%)	43 (61.4%)	198 (61.9%)
Out‐patient	95 (38%)	27 (38.6%)	122 (38.1%)
Average dose (mg/day)	480.6 (202)	462.8 (212)	476.7 (204.3)

† Major depressive disorder (MDD) diagnoses were available for 305 patients of whom 236 patients showed non‐significant and 69 patients significant body weight gain.

‡ PANSS: Positive and Negative Syndrome Scale, total scores (ranging from 30 to 210), with high scores indicating more severe psychopathology.

§ Defined as no prior exposure to antipsychotics lifetime.

**Figure 1:**
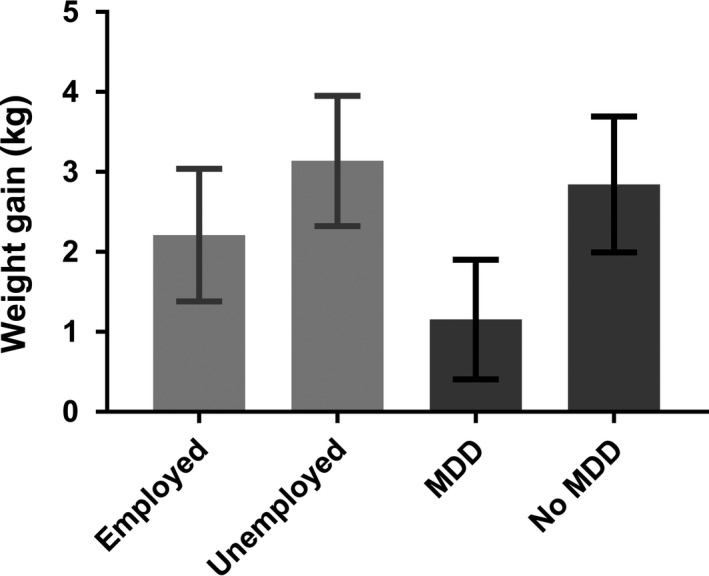
Amisulpride‐induced weight gain by employment status and current comorbid diagnosis of major depressive disorder (MDD) based on the results of the linear regression model. Data are shown as mean and standard deviations (whiskers).

The assumptions for linearity, normality, homoscedasticity and absence of multicollinearity were met in the linear regression model. Unemployment (*β* = 0.94, *P* = 0.016) positively contributed to AiWG, while age (*β* = −0.07, *P* = 0.031) and current comorbid major depression disorder (*β* = −1.6, *P* = 0.034) had a negative effect on AiWG (Table 2). Based on the results of the linear regression model, unemployed subjects showed an average AiWG of 3.14 kg (0.82), which was 1.4 times higher than in employed subjects: 2.21 kg (0.83) (Fig. 1). AiWG in patients without current comorbid MDD was 2.84 kg (0.85), which was almost 2.5 times higher than in those with MDD: 1.16 kg (0.75) (Fig. 1).

**Table 2:** Results of multivariable linear regression (light grey) and logistic regression models (dark grey). β coefficients for linear regression and odds ratios (OR) for logistic regression are provided with their corresponding 95% confidence intervals (CI) and p‐values. In bold are statistically significant (p=<0.05) findings

Predictor variables	Linear regression			Logistic regression		
	Outcome variable: Delta body weight			Outcome variable: Clinically relevant weight gain		
	*β*	95% CI	*P*‐value	OR	95% CI	*P*‐value
Sociodemographic variables						
Age (years)	**−0.07**	**−0.13, −0.01**	**0.031**	0.96	0.91, 1.01	0.106
Sex (male vs. female)	0.01	−0.89, 0.92	0.982	0.94	0.46, 1.89	0.852
Race (white vs. others)	0.16	−1.04, 1.35	0.798	1.34	0.52, 3.44	0.545
Unemployed (yes or no)	**0.94**	**0.18, 1.71**	**0.016**	**2.83**	**1.50, 5.36**	**0.001**
Baseline psychopathology associated variables						
Primary diagnosis (schizophreniform disorder vs. schizophrenia)†	0.59	−0.20, 1.37	0.141	**2.00**	**1.09, 3.68**	**0.025**
Current comorbid MDD (yes vs. no)†	**−1.61**	**−3.10, −0.12**	**0.034**	0.39	0.08, 1.79	0.224
PANSS total Scores‡	−0.01	−0.03, 0.01	0.380	0.99	0.97, 1.00	0.085
Treatment‐related variables						
Antipsychotic naïve (yes vs. no)	−0.50	−1.32, 0.33	0.235	0.72	0.37, 1.42	0.345
Type of care at baseline (in‐patient vs. out‐patient)	−0.27	−1.14, 0.60	0.537	1.34	0.67, 2.67	0.407
Average dose (mg/day)	0.00	−0.00, 0.00	0.824	0.99	0.99, 1.00	0.087
Baseline body weight (kg)	−0.02	−0.05, 0.01	0.182	**0.97**	**0.95, 0.99**	**0.032**

† According to the Mini International Neuropsychiatric Interview 5 plus.

‡ PANSS: Positive and Negative Syndrome Scale, total scores (ranging from 30 to 210), with high scores indicating more severe psychopathology.

Clinically relevant AiWG was observed in 70 (21.9%) patients. Logistic regression was performed to study the effect of the factors listed in Table 1 on clinically relevant AiWG. Unemployment and diagnosis of schizophreniform disorder showed a positive relationship with clinically relevant AiWG: unemployed patients had 2.8 times greater odds of clinically relevant AiWG than employed patients (OR 2.83, *P* = 0.001). Additionally, the odds of clinically relevant AiWG were 2 times greater in patients with a diagnosis of schizophreniform disorder than in patients with a diagnosis of schizophrenia or schizoaffective disorder (OR 2.0, *P* = 0.025). Those with relatively high body weight at baseline had smaller odds of suffering from clinically relevant AiWG (OR 0.97, *P* = 0.032).

## Discussion

We here characterized clinical and sociodemographic factors associated with amisulpride‐induced weight gain in a homogeneous, relatively large cohort (*n* = 305) of first‐episode psychosis patients following a month of treatment of amisulpride. We identify unemployment, young age, absence of current comorbid MDD, diagnosis of schizophreniform disorder, and lower body weight at baseline as factors positively associated with amisulpride‐induced weight gain.

Unemployment rates amongst patients with psychosis are often higher than 50% 22. This observation is also reflected in our current cohort where 56.3% of the patients were unemployed. We observed higher AiWG in unemployed patients using amisulpride than in employed participants of the trial. To our knowledge, this is the first study to report this association. In the general population, unemployment is a risk factor for body weight gain as it negatively affects food choices 17, 23. Similarly, low income due to unemployment could limit access to a healthy lifestyle (dieticians and sports facilities) 17, 23, in turn increasing the risk of weight gain. These indirect effects of unemployment have also been reported in schizophrenia patients 22 and may explain the accelerated weight gain detected here in unemployed participants. Another explanation for the high degree of AiWG observed in unemployed subjects could be a diminished sense of belonging, which may result in a passive lifestyle and thus increase chances of weight gain 18, 19. The relationship between MDD and weight gain may be explained by two phenomena. First, sedentary lifestyle due to fatigue and anhedonia may promote weight gain 17. Second, loss of appetite may trigger weight loss 24. The latter likely explains the lower body weight gain in subjects with MDD observed in our cohort. This is also in agreement with previous observations of weight loss rather than weight gain in depressed adolescents and adults 25. The negative association between age and AiWG observed in our cohort has previously been reported for other antipsychotics 6, 26, but not for amisulpride 8.

In the current cohort, patients with clinically relevant body weight gain had a lower baseline body weight. This finding has been reported by others and has been observed both in antipsychotic‐naïve and pre‐exposed patients 7, 8. Although the exact mechanism remains unknown, higher appetite levels and binge‐eating may explain part of this phenomenon 26.

While a positive association between symptom improvement and AiWG has been reported previously 27, we observed no correlation between change in PANSS scores and body weight change (Fig. S2). However, independent of other variables such as age and employment status, a higher percentage of the patients with ≥7% AiWG had been diagnosed with schizophreniform disorder, which is characterized by a shorter duration of symptoms and usually better prognosis than schizophrenia 28. We speculate that shorter duration of illness is associated with better treatment adherence and thus relatively high weight increases due to true amisulpride use. In support of this hypothesis, higher remission rates were indeed found in schizophreniform patients in the OPTiMiSE study 9.

Phenotypes previously associated with AiWG in response to other antipsychotics (e.g. antipsychotic‐naïve status, sex and race) were not associated with amisulpride‐induced weight gain in the current study. We speculate that differences in the pharmacological profiles 1 between second‐generation antipsychotics may underlie this observation.

## Strengths and limitations

Our understanding of AiWG especially in first‐episode psychosis patients is limited. Our first model (the linear regression model) is most informative because it uses weight as a continuous variable, while the second model (the logistic regression model) is directly applicable to clinical settings as this concerns clinically relevant AiWG. It additionally facilitates literature comparison 12 on weight gain with other antipsychotics. Despite clear advantages of our study design (e.g. the homogeneity and size of the cohort and the two statistical models providing consistent results), our results should be interpreted in light of some limitations. First, although comparing the weight‐inducing effects of amisulpride with an untreated control arm would have been ideal, matching these two groups on all sociodemographic factors would be impossible due to the unethical nature of withholding treatment. We cannot exclude other factors in addition to the use of amisulpride explaining a part of the increase in body weight. This constitutes a general drawback in single‐arm studies 29. Second, there is the issue of adherence introducing uncertainty in the reliability of the results, a recurring theme in many clinical trials. To address this uncertainty, we included type of care (in‐patient vs. out‐patient) as in‐patients are more likely to be treatment compliant but this proxy may not fully capture all variation in adherence. Finally, we have tried to incorporate as many clinical and sociodemographic variables as possible in our statistical models – variables hitherto not associated with AiWG and those previously associated with both AiWG and weight gain in the general population. However, possibly other relevant variables not measured in the OPTiMiSE trial may impact amisulpride‐induced weight gain too. For example, as income was not comprehensively assessed in OPTiMiSE, we cannot disentangle how this factor contributes to AiWG. In addition, genetic factors may also contribute to amisulpride‐induced weight gain, which is the subject of another study (manuscript in review). On a similar note, not all subgroups were equally represented, impacting statistical power for some of the variables we tested, such as race.

## Concluding remarks and future research

In sum, the current study is the first comprehensive study investigating clinical and sociodemographic factors associated with amisulpride use. The consistent association between unemployment and amisulpride‐induced weight gain across statistical models should prompt caution in clinicians prescribing amisulpride for this vulnerable patient population. Monitoring of this group is advisable to reduce chances of non‐adherence. Furthermore, implementation of timely weight management strategies may be considered in unemployed subjects to diminish the morbidity and mortality associated with metabolic syndrome. Similarly to the unemployed group, patients with schizophreniform disorder show a higher propensity to amisulpride‐induced weight gain relative to subjects with schizophrenia. They should therefore also be closely monitored. Finally, as predictors of AiWG may differ across antipsychotics, differences in susceptibility to AiWG between individuals and between antipsychotics should be further investigated in future genome‐wide studies.

## Funding

The OPTiMiSE trial was funded by the European Commission within the 7th Program (HEALTH‐F2‐2010‐242114). No additional funding was obtained for the current analyses.

## Conflicts of interest

BHE has received lecture fees from Otsuka Pharma Scandinavia AB and Lundbeck Pharma A/S. BVB became a full‐time employee at Novo Nordisk A/S after completion of the clinical study. All other authors declare that they have no conflicts of interest.

## Contributors

RP (MD, PhD) was involved in data analysis and interpretation and drafting of the article. DC (PhD) was involved in critical revision of the article including statistical methodology. StH (BSc) was involved in data analysis. CV (MD,PhD) and SG (MD, PhD) were involved in the interpretation and critical revision of the article. BHE (MD, PhD), BVB (PhD), PPGP (MD, PhD) and JB (MD, PhD) were involved in study conduct and critical revision. RSK (MD, PhD) obtained funding for OPTiMiSE and supervised the study. RSK and IWvR (PhD) designed the OPTiMiSE trial, participated in the data collection and critically reviewed the article. ADRH (MPhar, PhD) was involved in data interpretation and critical revision of the article. JJL (MD, PhD) was involved in the scientific design of the study, data interpretation and manuscript drafting and finalizing. All authors approved the final version to be published.

## Supporting information


**Table S1.** The number and percentage of participants per‐site included in the current study.
**Table S2**. Shift in BMI categories from inception to the end of trial, expressed as numbers and percentages of initial BMI categories: underweight (BMI<20); normal weight (BMI 20‐25); and overweight (BMI>25).
**Fig. S1.** Body weight gain (kg) across sites following 4‐week treatment of Amisulpride.
**Fig. S2.** A bivariate correlational analysis between change in total PANSS scores and body weight change *R* ^2^ = 0.002 and *P* = 0.415.

## Data Availability

Data used for the analyses are available upon reasonable request by sending in a research proposal to the OPTiMiSE steer group.
