# Supplementary material for: Phenotypic factors associated with amisulpride‐induced weight gain in first‐episode psychosis patients (from the OPTiMiSE cohort)
Source: Acta Psychiatr Scand. 2019 Jul 19;140(3):283–90. doi: 10.1111/acps.13074 (PMC6771865; doi:10.1111/acps.13074)
Supplement: Supplementary file 1 — Table S1. The number and percentage of participants per‐site included in the current study. Table S2. Shift in BMI categories from inception to the end of trial, expressed as numbers and percentages of initial BMI categories: underweight (BMI<20); normal weight (BMI 20‐25); and overweight (BMI>25). Fig. S1. Body weight gain (kg) across sites following 4‐week treatment of Amisulpride. Fig. S2. A bivariate correlational analysis between change in total PANSS scores and body weight change R 2 = 0.002 and P = 0.415. [file ACPS-140-283-s001.pdf]

Supplementary data ***Phenotypic factors associated with amisulpride-induced weight gain in patients with first-episode psychosis (from the OPTIMISE cohort)***

Pandit et al., Phenotypic factors associated with amisulpride-induced weight gain in patients with first-episode psychosis (from the OPTIMISE cohort)

Sites	Number of patients	Percentage of total (%)
A	2	0.6
B	2	0.6
C	11	3.4
D	6	1.9
E	29	9.1
F	6	1.9
G	10	3.1
H	50	15.6
I	32	10.0
J	17	5.3
K	31	9.7
L	19	5.9
M	9	2.8
N	11	3.4
O	11	3.4
P	14	4.4
Q	15	4.7
R	6	1.9
S	17	5.3
T	12	3.8
U	3	0.9
V	7	2.2
Total	320	100

Supplementary Table S1: The number and percentage of participants per-site included in the current study.

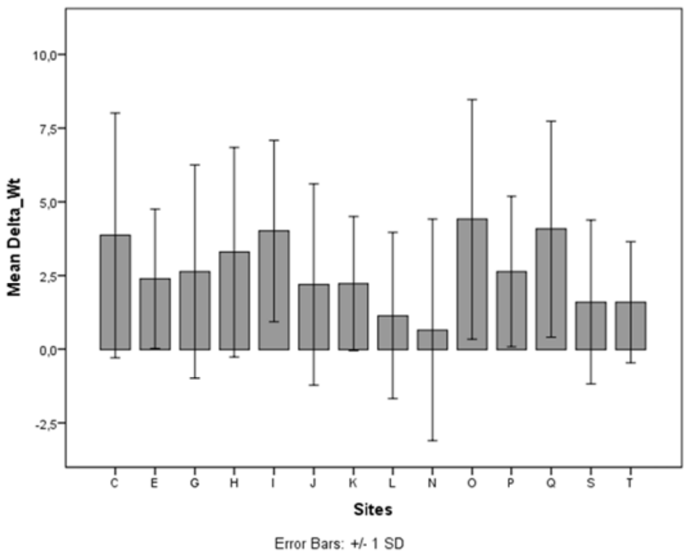

Supplementary Fig. S1: Body weight gain (kg) across sites following 4-week treatment of Amisulpride. Sites with less than 10 patients are not included in the graph. Data are shown as meanand standard deviations (whiskers).

		Study-end bodyweight		
		Underweight	Normal weight	Overweight
Baseline bodyweight	Underweight (26)	12 (46.2%)	14 (53.8%)	0
	Normal weight (214)	1 (0.5%)	189 (88.3%)	24 (11.2%)
	Overweight (80)	0	4 (5.0%)	76 (95.0%)

Supplementary Table S2: Shift in BMI categories from inception to the end of trial, expressed as numbers and percentages of initial BMI categories: underweight (BMI<20); normal weight (BMI 20-25) ; and overweight (BMI>25). For example**,** 26 patients were underweight at trial inception, 12 of whom (46%) remained underweight, 14 (54%) of whom reached a normal weight and none of whom became overweight at trial termination. Green: weight change might have a positive effect on health and well-being; yellow: weight change has neutral effects on health and well-being; red: weight change might have a detrimental effect on health and well-being.

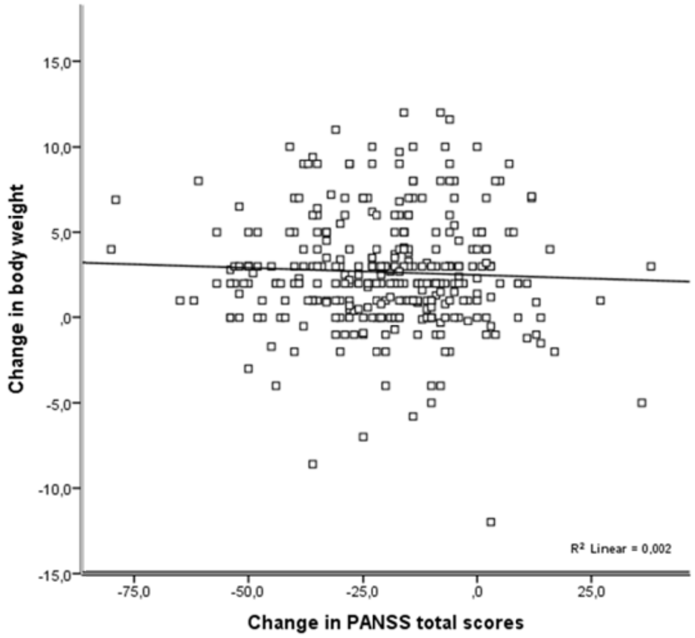

Supplementary Fig. S2: A bivariate correlational analysis between change in total PANSS scores and body weight change: R^2=0.002 and p=0.415.
